# SBS symptoms in relation to dampness and ventilation in inspected single-family houses in Sweden

**DOI:** 10.1007/s00420-017-1233-z

**Published:** 2017-06-17

**Authors:** Greta Smedje, Juan Wang, Dan Norbäck, Håkan Nilsson, Karin Engvall

**Affiliations:** 1 Department of Medical Sciences/Occupational and Environmental Medicine, Uppsala University, SE-751 85 Uppsala, Sweden; 2 School of Architecture and Built Environment/Division of Building Service and Energy, KTH Royal Inst of Technology, SE-100 44 Stockholm, Sweden

**Keywords:** Absolute air humidity, Concrete slab, Crawl space, Mould, SBS symptoms, *U* value

## Abstract

**Purpose:**

To investigate the relationships between symptoms compatible with the sick building syndrome (SBS) in adults and building dampness and ventilation in single-family houses.

**Methods:**

Within the Swedish BETSI study, a national sample of single-family houses were inspected by professional building experts, and adults living in the houses answered a questionnaire on SBS. Relationships between building factors and SBS were analysed using logistic regression.

**Results:**

Of the respondents, 23% reported having had weekly SBS symptoms during the last three months. A large proportion of houses exhibited building or construction problems. In total, 40% of houses had dampness problems in the foundation, and this was related to a higher prevalence of both mucous and dermal symptoms, and any SBS symptoms. Furthermore, high air humidity was related to more symptoms, with the relationship with absolute humidity being stronger than that with relative humidity or moisture load. Symptoms were also more prevalent in houses with a high *U* value, reflecting a poor thermal insulation. Compared to natural ventilation, living in a house with mechanical supply and exhaust ventilation was related to a lower prevalence of general symptoms and any SBS symptoms, but there were only weak associations between measured air exchange rate and symptoms.

**Conclusions:**

A large proportion of single-family houses have dampness problems in the foundation, and pollutants may enter the living space of the house and affect the health of the occupants. Furthermore, absolute air humidity should be measured more often in indoor air studies.

## Introduction

Different health symptoms may be related to a poor indoor environment. In the 1980s, the sick building syndrome (SBS) was defined as an increased prevalence of certain symptoms in groups of occupants of a building, and it was estimated that up to 30% of new and refurbished buildings were affected (WHO 1983, 1986).The SBS symptoms include non-specific symptoms from the eyes, upper airways and skin, as well as general symptoms such as headache and fatigue. Throughout the years, a number of studies investigated these symptoms and their relation to different indoor environmental factors, as well as to personal factors (Norbäck 2009). However, most studies were performed in workplaces, mainly offices, and only a few investigated the importance of the indoor environment of homes. In Sweden, the national ELIB (Energy Consumption in Buildings) survey was performed in the early 1990s and included data on SBS. It found that among adults living in multi-family houses, around 20% reported often having general symptoms such as headache, fatigue and nausea, and 15 and 8% reported mucosal and skin symptoms, respectively. The prevalences in single-family houses were around 10, 8 and 5%, respectively (Norlén and Andersson 1993). More recently, prevalence of symptoms was reported by adults living in multi-family houses in Stockholm, Sweden (Engvall et al. 2010), and a number of studies on SBS in relation to the home environment were performed in Japan. The prevalence of symptoms was reported to be between 10 and 19% (Araki et al. 2010; Takigawa et al. 2009, 2012). Still, there are few data on the prevalence of SBS among adults in single-family houses.

SBS is related to both personal and environmental factors (Norbäck 2009). Among the personal factors are gender, age, atopy and smoking (Engvall et al. 2010; Sahlberg et al. 2009). Among the environmental factors are building dampness and mould, volatile organic compounds and building ventilation.

Building dampness and mould are related to an increase of several diseases and symptoms of the airways and have also been related to both prevalence and incidence of SBS (Engvall et al. 2002; Gunnbjörnsdottir et al. 2003; Shoemaker and House 2006; Kanazawa et al. 2010; Sahlberg et al. 2010; Hulin et al. 2013). Dampness problems may occur for a number of reasons, including leakages from roofs and installations and ground water intrusion into the foundation. Different kinds of foundations of houses have been shown to be related to dampness problems (Mahooti-Brooks et al. 2004; Hägerhed-Engman et al. 2009; Toyinbo et al. 2016), but there are few studies on different kinds of constructions and their possible relationship with dampness-related SBS-type health effects, especially in adults. However, also other building factors have been suggested to increase SBS, such as volatile organic compounds and its sources including emissions from building materials, paint, etc. (Takigawa et al. 2012; Sahlberg et al. 2012, 2013).

Buildings are ventilated in order to provide fresh air and remove pollutants from the indoor environment. The importance of ventilation for SBS has been reviewed, and it has been concluded that sufficient air exchange is important in preventing SBS and should not be below approximately 0.5 air changes/h in dwellings. It is also important that the ventilation system is well maintained and designed to minimize pressure differences over the building envelope for preventing harmful pollutants to infiltrate indoors (Wargocki et al. 2002; Seppänen and Fisk 2004; Sundell et al. 2011). However, this evidence is partly indirect. Only a few studies have found a direct correlation between air exchange rate and health in homes. Furthermore, it has been discussed that current requirements for energy, perhaps resulting in tighter houses and reduced ventilation, may cause a deterioration of the indoor air quality with increased health problems as a result (Engvall et al. 2003; Bone et al. 2010).

Many epidemiological studies on SBS symptoms have gathered data on both health and exposure through occupant questionnaires and may suffer from reporting bias as regards exposure. In such studies, the possibilities to conclude on possible causal relationship are especially limited (Brauer et al. 2006). To assess the exposure by independent professionals might appear more reliable, but is also associated with various difficulties, apart from the major cost of investigating a large number of homes. Especially as regards dampness and mould, there is no golden standard for what to inspect or measure. A number of studies have tried to validate different methods, including inspection of building characteristics and dampness, and measurement of mould spores and mould-related compounds (Häverinen et al. 2001; Ren et al. 2001; Roussel et al. 2008; WHO 2009; Moularat et al. 2011; Reponen et al. 2010, 2013; Crawford et al. 2015). Several studies have shown a positive relationship between inspector-observed dampness problems and increased prevalence of health symptoms. However, the studies are still not conclusive in terms of how health-related dampness exposures should be identified. Thus, there is still a need to find which dampness indicators are related to health problems. The aims of the present study were to investigate:

- Prevalence of indoor environmental problems in single-family houses with a focus on building dampness and ventilation, assessed by professional inspectors.
- Relationship between SBS symptoms and inspected building dampness and ventilation of single-family houses.

## Materials and methods

### BETSI study

The BETSI (buildings, energy use, technical status and indoor environment) study was commissioned by The Swedish National Board of Housing, Building and Planning in 2006. The aim was to get representative information of the status of Swedish buildings, concerning certain parameters, as well as information on the relationships between health symptoms and the indoor environment. As regards dwellings, a cross section of buildings in Sweden was investigated using questionnaires, inspections and measurements. The selection of buildings was performed by Statistics Sweden. The buildings were selected by a multi-stage sampling procedure. In a first step, 30 municipalities, out of totally 290, were selected across Sweden through a stratified random selection taking into account geographic and demographic characteristics. The next step was selection of buildings, creating two samples, one consisting of single-family houses and one of multi-family houses. Data on all buildings and their construction year were obtained from the central building register in Sweden. Stratified random sampling was used to sample buildings based on the construction year in five classes (before 1960, 1960–1975, 1976–1985, 1986–1995 and 1996–2005) aiming to get the same number of buildings in each age class. Since most buildings in Sweden are old, there was an oversampling of new buildings. From these selected buildings, a subsample of buildings was chosen for inspections and technical measurements.

### Study population and questionnaire

The current study presents data from a subsample of 821 single-family houses chosen for inspections and technical measurements. The study population consist of all adults (≥18 years) living in these houses. Each adult received a questionnaire that included questions on health and personal factors. The questionnaire was developed at the Department of Medical Science, Uppsala University, based on previous studies. As an objective of the BETSI study was to compare the prevalence of SBS symptoms with the previous ELIB study, the same questions on symptoms were used. These questions originate from the MM-questionnaire which was developed at the Örebro University Hospital in Sweden, starting in the early 1980s. This questionnaire was tested for usefulness, reliability and validity and further developed on the basis of dozens of small pilot studies and information from external sources (Andersson et al. 1993; Andersson 1998). The questions on SBS symptoms have been used in a large number of studies, both in Sweden and internationally (Sundell and Lindvall 1993; Kemp et al. 1998; Mizoue et al. 2004; Sahlberg et al. 2012). The questions consist of an initial question: ‘During the last 3 months, have you had any of the following symptoms?’, followed by a list of symptoms, each with the possible responses ‘yes, often (every week)’, ‘yes, sometimes’, or ‘no, never’. The list of symptoms included three categories and a total of nine questions: general symptoms (fatigue; headache), mucous membranes symptoms (itching, burning, irritation of the eyes; irritated, stuffy or runny nose; hoarse, dry throat; cough) and dermal symptoms (dry or flushed facial skin; scaling, itching scalp or ears; dry, itching, red skin on hands). We also constructed a fourth category, any SBS symptoms, by combining the other three. Questionnaire data were collected in April–May 2008.

### Inspections and measurements

The houses were inspected by professional building experts according to a strict and comprehensive protocol developed by the National Board of Housing, Building and Planning, and all inspectors participated in a joint training before field work. Among aspects inspected were type of construction and fittings, general condition of the house and damages. Furthermore, drawings of the houses were checked for additional information of the construction, areas and volumes. The overall heat transfer coefficient (*U* value), reflecting the thermal transmittance and insulation, of the different construction materials and parts were noted, and the mean *U* value of the house was calculated. A lower *U* value means better thermal insulation.

During the inspection, moisture content in wood in the attic roof was measured as well as in the crawl space (if present), using a Protimeter Surveymaster^®^ moisture meter (General Electric Company, Connecticut, USA). Starting at the inspection, relative humidity and temperature were monitored indoors and outdoors during two weeks as well as the air exchange rate. Humidity and temperature were logged by a datalogger (Mitec Instrument, Säffle, Sweden). From these data, absolute humidity and moisture load were calculated. The air exchange rate was measured with measuring tubes using a tracer gas technique described in ISO 16000-8:2007. Perfluorocarbon tracer gas was used, and several tracer gas sources were positioned in the houses. The gas was collected passively in charcoal tubes for long-term averages according to the standard. Air exchange rates were calculated for different parts of the house as well as the mean of the building. Inspections and measurements were performed from October 2007–April 2008, during which houses usually were heated and window airing less frequent.

A number of damages and constructions at risk for damage were recorded by the inspectors. For the statistical analyses, we grouped them into problems from the foundation, outer walls and roof/attic, respectively. Problems that were recorded for each group are presented in Table 1.

**Table 1:** Grouping of inspected damages and risk constructions for building dampness

Foundation	*Houses with a concrete slab on the ground* Floor on wooden framework Drainage system not satisfactory
	*Houses with a crawl space* Visible water within the foundation Visible mould on surfaces Mouldy or other musty odour in the crawl space Moisture in wooden materials >14% Drainage system not satisfactory
	*Houses with a basement* Insulation and wood frame on the inside of outer wall Drainage system not satisfactory
Outer walls	Brick facade exposed to driving rain Unventilated and undrained outer walls with wooden frame and plaster
Attic and roof	Poor ventilation of attic Mouldy or other musty odour in the attic Visible mould on surfaces in attic Moisture in underlay wooden roof >14% Flat roof or roof that slopes inwards

### Statistical analysis

Statistical analyses were conducted using SPSS^®^ Statistics 22 (IBM^®^, New York, USA). Relationships between health symptoms and building factors were analysed using logistic regression, adjusting for personal factors that previous studies have shown are related to symptom reports such as gender, age and smoking. Associations were expressed as odds ratios (OR) with a 95% confidence interval (CI). Differences between groups were analysed by *X* ^2^ and ANOVA. In all statistical analyses, two-tailed analyses were applied.

## Results

In total, questionnaires were received from 1160 occupants living in 605 houses (74% of houses). Of these, 1097 occupants answered the questions on SBS symptoms, and 23% reported any such symptoms. Of the respondents, 49% were female and 51% were male, and mean age was 52.7 years, with a range from 18 to 89. Regular smokers were 7.5%. The prevalence of general symptoms was 17%, mucous symptoms 8.4%, dermal symptoms 6.3% and any SBS-type symptoms 23%. The prevalence of symptoms and personal factors are presented in Table 2. Symptom reports were more common in females than in males, and mean age was slightly lower among those reporting general and any SBS symptoms. However, age was not related to reporting mucous or dermal symptoms and smoking was not related to reporting symptoms.

**Table 2:** Symptoms and personal factors

	General symptoms		Mucous symptoms		Dermal symptoms		Any SBS symptoms	
	Yes	No	Yes	No	Yes	No	Yes	No
Percentage (number)								
Female	21 (112)	79 (424)	12 (65)	88 (469)	7.6 (40)	92 (484)	28 (152)	72 (384)
Male	13 (71)***	87 (484)	4.9 (27)***	95 (525)	5.1 (28)	95 (520)	18 (100)***	82 (457)
Smoker	17 (14)	83 (67)	11 (9)	89 (73)	3.8 (3)	96 (77)	23 (19)	77 (63)
Non-smoker	17 (169)	83 (835)	8.2 (82)	92 (917)	6.5 (64)	94 (923)	23 (231)	77 (774)
Mean (standard deviation)								
Age	46.0 (15.5)***	53.6 (15.5)	52.0 (16.7)	52.3 (15.7)	48.9 (15.9)	52.3 (15.7)	47.9 (16.2)***	53.7 (15.4)

***p value < 0.001 for difference between groups

The inspected houses’ mean year of construction was 1973 (SD 32), with a range from the 1600s to 2005 (md 1978). The prevalence of the different kinds of foundations was as follows: 45% had a concrete slab on the ground, 30% had a crawl space and 25% had a basement. As regards the type of ventilation system, 46% had natural ventilation, 36% mechanical exhaust system and 18% had a mechanical supply and exhaust system. Prevalence of building factors and problems are presented in Table 3. A large proportion of houses exhibited well-known building or construction problems, mainly concerning moisture problems. However, most of these houses did not have a mouldy odour at the time of inspection. Mean air exchange rate was often lower than the national guideline of at least 0.5 ac/h; 82% of buildings did not meet this recommendation. Furthermore, 7.9% did not meet the national recommendation of a moisture load not exceeding 3 g/m^3^. The *U* value decreased with year of construction (*p* < 0.000), with the newest buildings having about half the heat transmittance compared with houses build before 1960. However, there was also a substantial variation within each age group of buildings.

**Table 3:** Prevalence of specified dampness and building factors

	Yes		
Percentage (%) of houses with damages or risk constructions in/from, *n* = 605			
Foundation	40		
Outer walls	32		
Attic/roof	38		
Mould odour indoors	9.6		
Window pane condensation indoors	11		

	Mean	SD	Min–max
Moisture factor in wooden materials, mean, standard deviation (SD), and range			
Crawl space (%)	13	2.9	8 to 28
Attic (%)	13	2.8	7 to 28
Air humidity, mean, standard deviation (SD), and range			
Relative humidity (%)	34	6.2	19 to 57
Absolute humidity (g/m^3^)	6.4	1.1	4.0 to 10.8
Moisture load (g/m^3^)	1.7	0.9	−0.5 to 4.9
Ventilation and thermal transmittance, mean, standard deviation (SD), and range			
Air exchange rate, mean of house (ac/h)	0.36	0.18	0.07 to 1.14
Air exchange rate, largest bedroom (ac/h)	0.36	0.19	0.06 to 1.56
*U* value (W/m^2^ K)	0.49	0.21	0.20 to 1.43

Relationships between symptoms and building factors are given in Table 4. Air humidity, both relative humidity, absolute humidity, and moisture load, were found to be important factors, being related to both general and mucous symptoms, and accordingly, to reporting any SBS symptoms. Among the risk factors for building dampness in the construction, problems in the foundation were most important, being related to both mucous and dermal symptoms, and reporting any SBS symptoms. In total, 40% of all houses had dampness problems in the foundation. However, the prevalence of dampness-related risk factors varied between the different types of foundation. Of houses with a concrete slab, 24% had dampness-related damages or a risk construction in the foundation. Of houses with a crawl space the proportion was 43%, and of houses with a basement it was 51%. In Table 5, relationships between symptoms and the different types of foundations are presented. A concrete slab with dampness risk factors was related to a higher prevalence of symptoms, and a crawl space without dampness risk was related to a lower prevalence, compared to living in a concrete slab house without dampness risk factors in the foundation.

**Table 4:** Odds ratio (OR), confidence interval (CI) and *p* value for SBS symptoms in relation to dampness factors, adjusting for gender, age and smoking (*n* = 1097)

	General symptoms		Mucous symptoms		Dermal symptoms		Any SBS symptoms	
	OR (CI)	*p* value	OR (CI)	*p* value	OR (CI)	*p* value	OR (CI)	*p* value
Damages or risk construction in								
Foundation	1.15 (0.82–1.61)	0.418	1.99 (1.28–3.09)	0.002	1.95 (1.18–3.22)	0.009	1.58 (1.17–2.21)	0.002
Outer walls	1.27 (0.90–1.79)	0.183	1.55 (0.99–2.42)	0.053	1.45 (0.87–2.43)	0.155	1.20 (0.88–1.63)	0.250
Roof/attic	1.14 (0.79–1.53)	0.488	1.29 (0.80–2.06)	0.294	0.97 (0.56–1.69)	0.914	1.19 (0.87–1.64)	0.277
Moisture in wooden materials								
Crawl space^a^	0.98 (0.87–1.10)	0.705	1.00 (0.85–1.19)	0.965	1.09 (0.93–1.28)	0.310	0.99 (0.89–1.10)	0.852
Attic^a^	1.01 (0.94–1.08)	0.803	1.10 (1.02–1.19)	0.016	1.08 (0.99–1.17)	0.100	1.02 (0.96–1.08)	0.540
Mould odour indoors	1.57 (0.92–2.68)	0.102	1.49 (0.75–2.94)	0.253	1.05 (0.44–2.52)	0.911	1.61 (1.00–2.60)	0.050
Window pane condensation	1.05 (0.62–1.77)	0.868	1.41 (0.75–2.64)	0.289	2.19 (1.14–4.18)	0.018	1.47 (0.95–2.28)	0.085
Air humidity								
Relative humidity^b^	1.38 (1.06–1.82)	0.018	1.36 (0.95–1.93)	0.094	1.39 (0.92–2.08)	0.119	1.40 (1.09–1.79)	0.007
Absolute humidity^c^	1.29 (1.10–1.51)	0.002	1.29 (1.04–1.59)	0.018	1.18 (0.93–1.50)	0.182	1.25 (1.09–1.45)	0.002
Moisture load^c^	1.31 (1.09–1.59)	0.005	1.28 (0.99–1.65)	0.054	1.19 (0.89–1.60)	0.231	1.20 (1.01–1.42)	0.042

^a^Odds ratio per 1% point change of moisture content

^b^Odds ratio per 10% points change of relative humidity

^c^Odds ratio per 1 g/m^3^ change

**Table 5:** Odds ratio (OR), confidence interval (CI) and *p* value for SBS symptoms in relation to dampness factors in different kinds of foundations, adjusting for gender, age and smoking (*n* = 1097)

	General symptoms		Mucous symptoms		Dermal symptoms		Any SBS symptoms	
	OR (CI)	*p* value	OR (CI)	*p* value	OR (CI)	*p* value	OR (CI)	*p* value
Crawl space with risk factor	1.21 (0.72–2.04)	0.475	1.34 (0.68–2.64)	0.396	1.47 (0.69–3.14)	0.317	1.45 (0.92–2.31)	0.112
Crawl space without risk factor	0.57 (0.33–1.01)	0.052	0.35 (0.13–0.92)	0.033	0.36 (0.12–1.07)	0.067	0.56 (0.34–0.94)	0.027
Basement with risk factor	1.39 (0.80–2.40)	0.240	1.46 (0.72–2.93)	0.291	1.99 (0.94–4.20)	0.070	1.54 (0.95–2.51)	0.082
Basement without risk factor	1.59 (0.96–2.61)	0.070	1.03 (0.50–2.14)	0.929	0.91 (0.38–2.18)	0.826	1.64 (1.04–2.59)	0.033
Concrete slab with risk factor	1.20 (0.67–2.12)	0.542	2.19 (1.14–4.19)	0.018	1.65 (0.75–3.61)	0.215	1.82 (1.11–2.97)	0.017
Concrete slab without risk factor	1		1		1		1	

Compared to living in houses with natural ventilation, living in a house with a mechanical supply and exhaust ventilation system was related to a lower prevalence of general symptoms, and any SBS symptoms. Houses with a mechanical supply and exhaust ventilation system had a higher mean air rate than those with other types. Still, 70% of these houses did not meet the standard of 0.5 ac/h, compared to 87 and 88% of houses with natural ventilation and mechanical exhaust ventilation, respectively. Symptoms were not significantly related to mean air exchange of the house. However, both mucous and dermal symptoms were less prevalent among those living in a house with a higher air exchange rate in the largest bedroom. Those living in buildings with a higher *U* value reported more general symptoms, and any SBS symptoms (Table 6).

**Table 6:** Odds ratio (OR), confidence interval (CI) and *p* value for SBS symptoms in relation to ventilation and thermal transmittance, adjusting for gender, age and smoking (*n* = 1097)

	General symptoms		Mucous symptoms		Dermal symptoms		Any SBS symptoms	
	OR (CI)	*p* value	OR (CI)	*p* value	OR (CI)	*p* value	OR (CI)	*p* value
Ventilation								
Natural ventilation	1		1		1		1	
Mechanical exhaust	0.96 (0.67–1.37)	0.810	0.84 (0.52–1.36)	0.478	1.05 (0.61–1.80)	0.867	0.85 (0.62–1.17)	0.325
Mechanical supply and exhaust	0.54 (0.33–0.90)	0.017	0.58 (0.30–1.14)	0.116	0.57 (0.25–1.27)	0.169	0.60 (0.40–0.93)	0.020
Air exchange rate, mean of house^a^	0.98 (0.89–1.08)	0.677	0.89 (0.77–1.02)	0.101	0.87 (0.74–1.03	0.108)	0.93 (0.85–1.02)	0.106
Air exchange rate, largest bedroom^a^	0.96 (0.88–1.06)	0.425	0.84 (0.73–0.98)	0.025	0.40 (0.32–0.96)	0.039	0.92 (0.84–1.00)	0.050
Thermal transmittance								
*U* value^b^	1.10 (1.02–1.19)	0.010	1.01 (0.91–1.13)	0.788	0.95 (0.84–1.08)	0.463	1.08 (1.01–1.15)	0.033

^a^Odds ratio per 0.1 change of air exchange rate

^b^Odds ratio per 0.1 change of *U *value

Although different constructions may be typical for certain time periods, we did not find any relationship between age of building and health symptoms.

## Discussion

We found significant relationships between SBS-type symptoms in adults and the occurrence of several building factors that is usually regarded as problematic. Thus, the study could confirm that these problems also affect SBS-type symptoms in the occupants.

In this study, 821 single-family houses were chosen and the study population consisted of all adults living in these houses. However, we do not have access to how many they were; thus we cannot calculate an exact response rate of the questionnaire. In total, questionnaires were received from 1097 occupants living in 605 houses (74% of houses), corresponding to a mean of around 1.8 respondents per house. In Sweden, mean number of adults per single-family house is around 2.2. Overall, this might indicate a response rate of around 65%. Nowadays, questionnaire studies in Sweden tend to have a responses rate of around 50%. A slightly higher response rate in our study may reflect a special interest among house owners for issues related to their dwelling. Building factors in randomly chosen houses were assessed and measured by professional building inspectors through a strict protocol, and without knowledge of the occupants’ health status. Furthermore, data on health symptoms were collected independent of the inspection. Thus, the risk of selection or information bias should be low. The weaknesses of the study rather lie in the cross-sectional design and its inherent limitation when it comes to understanding causality, and the multiple building factors recorded, leading to multiple statistical analyses and a relatively low statistical power. A longitudinal study focusing on reported associations would be valuable.

A major aim of the study was to investigate what building dampness problems that can be observed during a non-destructive inspection were related to health symptoms. In order to do so, we constructed dampness indexes for the different parts of the building (foundation, outer walls, attic/roof). Each index contained data on three different levels of dampness problems: measured dampness, observed dampness and dampness risk constructions. We found that these indices could indicate buildings having foundations with health-related problems. However, it would be important to further elaborate the usefulness of such tools by analysing the different levels of dampness problems separately. Unfortunately, this was not possible in our study, due to lack of statistical power.

For houses with a concrete slab or crawl space, the factors included in the inspection of building dampness risks were successful in discovering different relationships to health in houses with a foundation that was classified as having problems compared to those without problems. However, for houses with a basement, there was a tendency of a higher prevalence of symptoms, regardless of the dampness risk classification. For basements, the dampness index included two questions; ‘insulation and wood frame on the inside of the outer wall’ and ‘drainage not satisfactory’. Although these are important risk factors, constructions below ground level may be associated with even more problems, not covered by the present index.

The study confirms that within a certain type of construction, there might be different details that are important for dampness conditions and health. This is in accordance with a previous Swedish study (Hägerhed-Engman et al. 2009) in which problems were found for houses with a concrete slab foundation, but only in buildings built before 1983, which would reflect that in older houses insulation was often placed above the slab, which is a well-known risk construction. In recent years, the crawl space has been discussed as a risk construction, but our study found that also for the crawlspace there were houses without dampness problems and the occupants in these houses had the lowest prevalence of SBS symptoms. It would be important to further investigate the characteristics of these crawl spaces and houses. Furthermore, these findings indicate that in a climate with relatively cold and dry air such as in Sweden, dampness in the foundation might be the most important problem. The results also confirm that building dampness problems that are important to health do not need to occur in the living space of the house. Due to air movements within the building, polluted air is transported from affected areas into the living space, including from the basement (Miranda et al. 2011; Du et al. 2015).

It is well known that in indoor environments with high air humidity, the presence of mould and house dust mites is increased, as well as the prevalence of e.g. respiratory symptoms (WHO 2009). In our study, we used three measures of air humidity (RH, AH, moisture load). All were positively related to symptoms, but the statistical analyses tend to indicate that the relationship was strongest for absolute humidity. Relative humidity is the most commonly used air humidity variable in epidemiological studies. Our study supports the conclusions of a recent review (Davis et al. 2016) that stresses the importance of choosing an air humidity variable that is relevant according to the medical outcome under study and recommends a measure of moisture content (e.g. AH) to be used in most cases.

There was no relationship between SBS-type symptoms and the mean air exchange rate of the house. However, the air exchange rate might differ between rooms, and we found a relationship between symptoms and the air exchange of the largest bedroom. Of the different rooms in a dwelling, most occupants spend the largest amount of time in their bedroom, thus air exchange of the bedroom would be especially important. We do not know which bedroom those who answered the questionnaire usually slept in, but in Sweden it is common that in families with children the parents sleep in the largest bedroom.

The thermal transmittance of the house was related to general symptoms. As expected, the *U* value was generally lower in newer houses, but analyses controlling for age of building showed similar results (data not shown). In fact, there was a substantial variability of *U* values within different age groups of buildings. This indicates that the *U* value itself is the important factor related to health symptoms, and is not just a proxy for year of construction. A low *U* value is typical of energy-efficient buildings. Occasionally it has been suggested that energy efficiency in buildings would result in poor indoor air quality and health problems (Sharpe et al. 2015). Our study does not confirm such an assumption. On the contrary, our study indicates a lower prevalence of SBS-type symptoms in buildings with a low thermal transmittance.

In total, 17% reported general symptoms, 8.4% mucous symptoms and 6.3% skin symptoms. These prevalences may be compared with those from the previous national Swedish survey ELIB, performed in the early 1990s (Norlén and Andersson 1993). For mucous and skin symptoms, the prevalences are very similar. However, for general symptoms there is an increase, especially for ‘fatigue’. In the present study, 15% reported often being tired, while in the former it was 9%. The reason for this increase remains unclear. Very few respondents answered that they thought their symptoms were due to the indoor environment at home, that few it was not possible to perform any analyses on the possible relationship between such attributed symptoms and housing conditions. However, we found relationships between symptoms and environment, even though the occupants themselves did not make such attributions. Thus, occupant complaints may not be a sufficient aspect when assessing health-related indoor environmental problems.

This study demonstrates relationships between the presence of dampness problems in the home and the health of the residents. To evaluate the effect on health of remediation of such dampness problems should be important. However, there are relatively few intervention studies that sought to investigate this issue. In a Cochrane review, the literature is summarized by Sauni et al. (2015). They found no or moderate effects of the remediation on health and concluded that better studies are needed. Most previous intervention studies have focused on the effects on asthma and lower respiratory symptoms, but also effects on the kind of symptoms we have studied here, including general, dermal and upper respiratory symptoms, need to be evaluated.

## Conclusions

Dampness-related damages and risk constructions in single-family houses assessed by professional inspectors were related to SBS-type symptoms in the occupants, especially dampness problems in the foundation. Thus, pollutants from the foundation may enter the living space of the house and affect indoor air quality. Furthermore, high air humidity was related to more symptoms, with the relationship with absolute humidity being even stronger than that with relative humidity or moisture load. This indicates that absolute humidity should be measured more often in indoor air investigations.
